# Identification of Growth-Related Key Genes Based on Nonlinear Fitting of Weight Growth Curves in Min Pigs

**DOI:** 10.3390/ani16172783

**Published:** 2026-09-04

**Authors:** Zhenxing Zhou, Yi Liu, Xinning Zhang, Li Wang, Shiquan Cui, Shengwei Di, Yuan Xu, Xibiao Wang

**Affiliations:** College of Animal Science and Technology, Northeast Agricultural University, No.600 Changjiang Road, Xiangfang District, Harbin 150030, China; zzx6954@163.com (Z.Z.); s240502102@neau.edu.cn (Y.L.); s240502104@neau.edu.cn (X.Z.); wanglisy1982@126.com (L.W.); cuishiquan@neau.edu.cn (S.C.); dishengwei@neau.edu.cn (S.D.)

**Keywords:** min pig, growth curve, nonlinear mixed-effects model, logistic model, transcriptome analysis

## Abstract

The Min pig, a Chinese indigenous breed, is valued for its excellent meat quality and stress tolerance. Nevertheless, its growth rate falls considerably short of commercial pig breeds, a pattern typical of most unselected indigenous populations, and marked individual variation further undermines its economic viability. To explore the genetic basis of this variation, we evaluated a series of nonlinear fixed-effects and mixed-effects models based on the Gompertz, Logistic, and von Bertalanffy functions using body weight records from 92 Min pigs. Model selection was based on AIC, BIC, and leave-one-out cross-validation. The best-fitting model was a Logistic nonlinear mixed-effects model with individual-level random effects on all three growth parameters (Asymptotic weight, timing parameter, and be parameter), which clearly outperformed models with simpler random-effect structures and fixed-effects models. From this model, we identified three characteristic growth transition points: the early transition point (Growth Rate Index, GRI) at 94.83 days (22.32 kg), the single inflection point (Maximum Growth Rate, MGR) at 166.06 days (52.80 kg), and the late transition point (Late Growth Rate Index, LGRI) at 237.29 days (83.29 kg), with a maximum absolute growth rate of 488.2 g/day. These points partitioned growth into initial acceleration, rapid growth, deceleration, and Asymptotic growth phases. Using individual fitted growth curves, we selected five fast-growing and five slow-growing pigs that reached approximately 90 kg during the plateau phase, defined as a predicted body weight of at least 95% of the individual Asymptotic weight. The fast-growing group reached 90 kg at 240.5 ± 20.42 days, whereas the slow-growing group reached the same weight at 286.67 ± 19.20 days. At the 90 kg slaughter weight, we collected longissimus dorsi muscle samples from these pigs during the plateau phase and performed RNA-seq. Transcriptome analysis revealed 864 differentially expressed genes between the two groups, with 540 upregulated and 324 downregulated in the fast-growing group. Pathway enrichment implicated the PI3K-Akt and TGF-β signaling pathways in muscle development, and differential expression of IGFN1, DCN, COL3A1, MYOC, COL1A2, COL1A1, and IGF2 may explain the growth variation between the groups. In summary, the Logistic mixed-effects model with individual-level random effects on all growth parameters effectively captures the growth pattern of Min pigs, and the PI3K-Akt and TGF-β pathways likely mediate growth differences in this breed.

## Simple Summary

The Min pig, a Chinese indigenous breed, is prized for its superior meat quality and stress resistance. However, its growth rate lags well behind commercial pig breeds, and substantial individual variation further constrains its industrial value. To define the growth pattern of this breed, we fitted nonlinear models to reconstruct its growth trajectory and pinpoint critical inflection points of growth rate change. We also compared the longissimus dorsi muscle transcriptomes of fast- and slow-growing pigs, which revealed key genes and pathways linked to growth variation. Together, these results clarify the distinctive growth traits of Min pigs and the molecular drivers of their growth differences, offering a basis for genetic improvement programs.

## 1. Introduction

The Min pig is an indigenous breed native to Northeast China that exhibits a combination of desirable traits. One of its most notable traits is excellent maternal performance, which is reflected in the significantly lower piglet crushing mortality during lactation reported by Huang et al. (2021, 2025) [1,2]. Building on this, the breed shows strong stress resistance, with Wang et al. (2025) finding that Min piglets are particularly resistant to common intestinal diseases such as diarrhea during lactation and weaning [3]. Supporting this, Chen et al. (2022) linked specific SNP loci in the STAT3 gene to this enhanced diarrhea resistance at the molecular level [4]. Beyond its robust health and reproductive performance, Min pigs are well-regarded for their superior meat quality, which includes bright red muscle color, desirable flavor, and high intramuscular fat content, as noted by Jin et al. (2024) [5]. These favorable traits largely stem from long-term domestication and conservation under traditional extensive feeding systems, which have maintained these traits without intensive artificial selection for growth.

Growth performance is one of the most valued traits in pig production. Like most Chinese indigenous pig breeds, the Min pig has not undergone intensive selection for growth traits, resulting in a considerable growth performance gap compared with introduced commercial pig breeds. Wang et al. (2024) reported a daily weight gain (DWG) of 430 g for Min pigs during the growing–finishing period [6]. Pu (2023) and Qian (2016) reported DWG values of 518 g for Meishan, 433 g for Laiwu, and 392.5 g for Ningxiang pigs, respectively [7,8]. All of these values are substantially lower than those of commercial pig breeds, such as Landrace (816.9 g), Yorkshire (842.3 g), and Duroc (1079 g) [9,10,11]. The absence of targeted selection in growth performance has also led to considerable individual variation within indigenous breeds. For instance, Lu (2024) observed significant differences in growth and fattening traits among Meishan pigs, a pattern also observed in our Min pig population [12]. Such large individual variation hinders comparative analyses of gene expression profiles between phenotypically distinct individuals at the same growth stage, thereby limiting the precise identification of genetic mechanisms underlying growth traits in indigenous pigs. This phenotypic variation is likely driven by differential expression of key candidate genes involved in growth regulation. However, the molecular mechanisms underlying growth performance in Min pigs remain largely unexplored, and it is unclear how these favorable traits coexist with the absence of selective pressure for rapid growth. Among the genes that may contribute to this variation, *IGF2* is of particular interest, a well-characterized regulator of growth in pigs whose role in promoting muscle development is well established in commercial pig breeds [13,14,15,16]. However, its expression patterns and regulatory mechanisms in indigenous Chinese breeds, particularly in Min pigs, remain poorly understood. Given that reliable molecular comparisons require samples from comparable developmental stages, a robust method for growth stage classification is needed before such analyses can be conducted.

To attain the stated aim, a clear understanding of animal growth and development patterns is essential [17,18]. Growth curve fitting has emerged as a robust approach for investigating porcine growth trajectories [19]. Currently, the main models for fitting growth curves in livestock and poultry include nonlinear fixed-effects models and nonlinear mixed-effects models [20,21]. Mixed-effects models can be further divided into single-level and multi-level variants, depending on the number of hierarchical levels or factors incorporated [22]. When population-level effects such as litter, batch, or sex are present and not confounded with other factors, including them as random effects may improve model fit by accounting for both individual heterogeneity and inter-population variability. In recent years, multi-level nonlinear mixed-effects models have attracted increasing attention. For example, Strathe et al. (2010) constructed four multi-level nonlinear mixed-effects models (Gompertz, Logistic, Bridges, Lopez) to assess weight gain in commercial pigs and found that the Lopez model—which accounted for individual, litter, sex, and data autocorrelation—provided the best fit [23]. Similarly, Zhang et al. (2012) identified the Richards two-level nonlinear mixed-effects model, incorporating individual, sex, and data autocorrelation, as the optimal choice for describing the growth curve of Muscovy ducks [24]. Furthermore, multi-level nonlinear mixed-effects models have been extended to pharmacokinetic modeling, where they serve as a powerful tool for exploring the effects of covariates such as age, body weight, and disease status on drug efficacy [25,26].

To date, no systematic comparison between nonlinear fixed-effects models and nonlinear mixed-effects models with alternative random-effect structures has been conducted specifically for Min pig growth curve fitting. Therefore, the objectives of this study were threefold: (1) to identify the optimal model for fitting Min pig growth curves by comparing nonlinear fixed-effects models and nonlinear mixed-effects models that differed in their random-effect specification, using the Gompertz, Logistic, and von Bertalanffy functions; (2) to characterize the body weight growth trajectory of Min pigs based on the best-performing model; and (3) to investigate gene expression profiles in the longissimus muscle of pigs with divergent growth rates, with the aim of exploring the genetic basis underlying individual variation in growth traits.

## 2. Materials and Methods

### 2.1. Experimental Animals

The Min pig, a typical indigenous breed from northern China, has a completely black coat, medium body size, large drooping ears, thick and wrinkled skin, dense winter underfur, and seven or more pairs of teats. This breed exhibits high reproductive performance, strong stress resistance, and excellent meat quality. We obtained the experimental pigs from the Lanxi County Breeding Farm, a national-level conservation farm in Suihua City, Heilongjiang Province. The animals came from two batches: 51 pigs (27 gilts and 24 boars) born in late December 2021, and 41 pigs (22 gilts and 19 boars) born in late March 2022. Within each batch, birth dates were similar. Male piglets were castrated at 35 days of age, and all piglets were weaned at 45 days. Both batches received identical feeding and management throughout the study.

### 2.2. Data Collection

Growth curves were fitted using body weight data from 92 Min pigs. For the first batch, weights were recorded at 14 time points: 100, 115, 130, 145, 160, 175, 190, 205, 220, 235, 250, 265, 280, and 295 days of age. For the second batch, we measured weights at 45, 75, 100, 115, 130, 145, 160, 175, 190, 205, 220, 235, 250, and 265 days of age.

### 2.3. Growth Curve Fitting

#### 2.3.1. Nonlinear Model Construction

We constructed three nonlinear fixed-effects models, using the Gompertz [27], Logistic [28], and Von Bertalanffy [29] functions, respectively. The mathematical expressions are as follows:

$$y=kexp\left(-aexp\left(-bt\right)\right);$$

$$y=\frac{k}{1+aexp\left(-bt\right)};$$

$$y={k\left(1-aexp\left(-bt\right)\right)}^{3};$$

Among them, k represents the maximum mature weight, a represents the time-dependent parameter, and b represents the maturity rate.

To account for repeated measurements within individual pigs, nonlinear mixed-effects models were fitted using the general form.

$$y_{it}=f({Age}_{it};\theta_{i})+\varepsilon_{it};$$

where $y_{it}$ is the observed body weight of pig i at messurement time t, and $f\left(.\right)$ denotes the nonlinear growth function (Gompertz, Logistic, or von Bertalanffy). The individual-specific parameter vector $\theta_{i}={\left(K_{i},a_{i},b_{i}\right)}^{T}$ was decomposed as

$$\theta_{i}=\beta +b_{i};$$

where β represents the population-level fixed effects and $b_{i}$ represents the individual-level random effects. The random effects were assumed to follow a multivariate normal distribution with mean zero and an unstructured variance–covariance matrix Ψ, allowing correlations among the random effects:

$$b_{i}∼N(0,\Psi );$$

The residual errors $E_{it}$ were assumed to be independent and normally distributed with constant variance:

$$E_{it}∼N(0,\sigma^{2});$$

In the final model, random effects were included on all three growth parameters (K, a, and b), providing individual-specific estimates of the entire growth trajectory.

#### 2.3.2. Fit Test

Model goodness of fit typically varies among populations. Therefore, fitting the growth curve of a specific population often requires fitting multiple nonlinear models and selecting the optimal one based on goodness-of-fit tests and other validation approaches. In this study, we used the Akaike information criterion (AIC), Bayesian information criterion (BIC), and log-likelihood (logLik) to evaluate model fitting performance. The calculation formulas are as follows [30,31]:

$$AIC=n\ln\left(\frac{SSE}{n}\right)+2k$$

$$BIC=n\ln\left(\frac{SSE}{n}\right)+k\ln n$$

$$logLik=-0.5\left(n\log2\pi +n\log{(sigma}^{2})+\frac{SSE}{{sigma}^{2}}\right)$$

Among them, n represents the number of observed values, SSE represents the sum of squared residuals, k represents the number of parameters, and sigma2 represents the variance estimate. According to the expression, the smaller the AIC and BIC values, and the larger the logLik value, the higher the fitting degree.

#### 2.3.3. Model Diagnosis and Validation

Model adequacy was assessed using residual diagnostics and leave-one-out cross-validation (LOOCV). Standardized residuals were examined for normality (Q–Q plot; Shapiro–Wilk test), heteroscedasticity (residuals vs. fitted values and age), and autocorrelation (ACF). We also compared the final model with extended models incorporating a continuous-time autoregressive correlation structure (corCAR1) and a power variance function (varPower); these were evaluated using BIC. Prediction performance was assessed by LOOCV, in which one entire pig was excluded per iteration and predictions were generated using only the fixed-effects component. Prediction accuracy was summarized by RMSE (root mean squared error) and MAPE (mean absolute percentage error). The mathematical expressions are as follows:

$$r_{ij}=\frac{e_{ij}}{\hat{\sigma }\sqrt{1-h_{ij}}}$$

Among them, ${℮}_{ij}$ represents the raw residual, $\hat{\sigma }$ represents the estimated residual standard deviation, and $h_{ij}$ represents the leverage value.

$$RMSE=\sqrt{\frac{1}{n}\sum_{i=1}^{n}(y_{i}-{\hat{y}}_{i}{)}^{2}}$$

$$MAPE=\frac{100\%}{n}\sum_{i=1}^{n}\left|\frac{y_{i}-{\hat{y}}_{i}}{y_{i}}\right|$$

Among them, $y_{i}$ represents the observed value and $\hat{y_{i}}$ represents the leave-one-out predicted value.

#### 2.3.4. Derivation of Growth Characteristic Points

From the estimated growth curve parameters, we derived one inflection point and two characteristic transition points. The single inflection point, termed the Maximum Growth Rate (MGR), corresponds to the age at which absolute growth rate reaches its maximum. The early transition point, termed the Growth Rate Index (GRI), is defined as the age at which the second derivative of the growth function reaches its maximum, marking the transition from increasing to decreasing acceleration. The late transition point, termed the Late Growth Rate Index (LGRI), is defined as the age at which the second derivative reaches its minimum, marking the transition from rapid to slow deceleration. Table 1 lists the formulas used to calculate the ages and body weights corresponding to GRI, MGR, and LGRI, as well as the maximum absolute growth rate.

### 2.4. Statistical Analysis

We compiled and preprocessed Min pig body weight data in Microsoft Excel. At each age, weight records deviating by more than ±3 standard deviations from the mean were removed, resulting in the exclusion of 18 weight records (1.5% of the total data). Subsequently, we constructed nonlinear fixed-effects models using the nls() function in R (version 4.3.0) and two-stage nonlinear mixed-effects models using the nlme() function from the nlme package (version 3.1-162). Model goodness-of-fit was evaluated using AIC, BIC, and log-likelihood values calculated via the AIC(), BIC(), and logLik() functions in R. Data visualization, including scatter plots, was performed using the ggplot2 package (version 3.4.2).

From the fitted growth curve, we identified three characteristic transition points based on the derivatives of the growth function. These three points partitioned the growth trajectory into four successive phases: initial acceleration (from the onset of growth to GRI), rapid growth (from GRI to MGR), deceleration (from MGR to LGRI), and Asymptotic growth (after LGRI), during which growth rate declined progressively and body weight approached its Asymptotic value. The ages and body weights corresponding to GRI, MGR, and LGRI were derived from the final population-level parameter estimates.

### 2.5. Transcriptome Sequencing and Analysis

#### 2.5.1. Sample Selection

**Figure A1:**
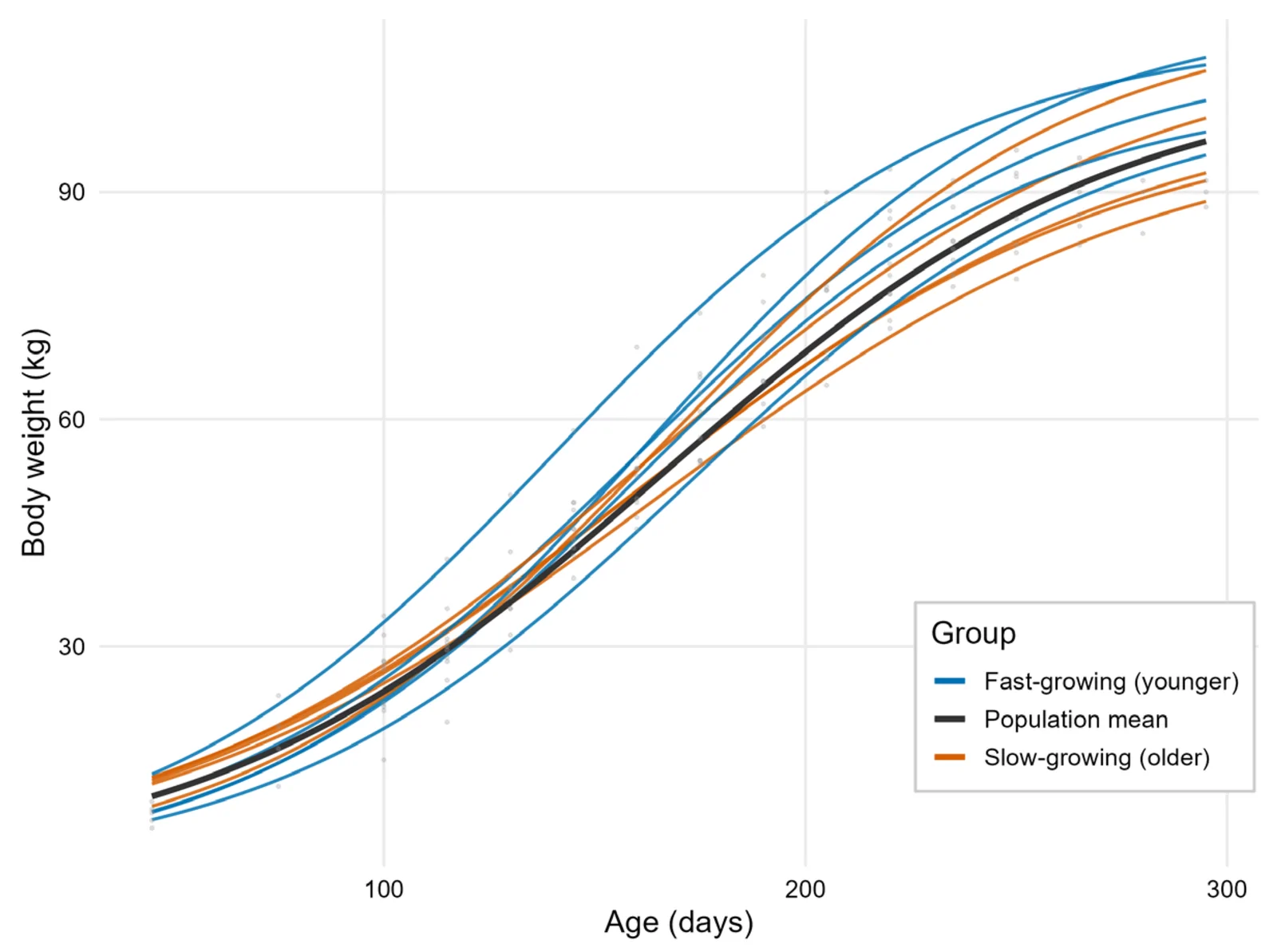
Individual growth curves for the ten selected pigs used in transcriptome analysis. Fitted curves were derived from the final Logistic nonlinear mixed-effects model with individual-level random effects on K, a, and b. The population mean curve is shown in black; fast-growing pigs (younger at slaughter) are shown in blue, and slow-growing pigs (older at slaughter) are shown in orange. Observed body weight records are indicated by semi-transparent points.

Table 2 summarizes the transcriptome samples used for growth rate analysis. All samples were collected from sows during the asymptotic growth phase. We selected 10 individuals with distinct slaughter ages: 5 fast-growing pigs (younger at slaughter) and 5 slow-growing pigs (older at slaughter). Transcriptome samples were obtained from the longissimus dorsi muscle at the thoracolumbar junction. Data are expressed as mean ± standard deviation (SD). Between-group differences in slaughter age were assessed using an independent samples Student’s *t*-test (two-tailed). Prior to the test, normality (Shapiro–Wilk test, both groups *p* > 0.05) and homogeneity of variances (Levene’s test, *p* > 0.05) were confirmed. The slow-growing group had a significantly higher slaughter age (286.67 ± 19.20 days) than the fast-growing group (240.50 ± 20.42 days), with a mean difference of 46.17 days (t = 3.682, df = 8, two-tailed *p* = 0.006). Individual growth curves for the ten selected pigs are shown in Appendix A Figure A1.

#### 2.5.2. RT qPCR Validation

To validate the RNA-seq data, we selected GAPDH as the reference gene and randomly chose seven differentially expressed genes (DEGs) for quantitative real-time PCR (RT-qPCR) validation based on their functional relevance to muscle development and growth regulation, as well as their differential expression patterns (|log2FC| ≥ 1.5 and *p* < 0.05). We designed primers using Primer Premier 5.0 and had them synthesized at Heilongjiang Jianshu Gene Technology Co., Ltd., Beijing, China (primer sequences in Table 3).

For RT-qPCR, we reverse-transcribed total RNA from the same longissimus dorsi muscle samples used for RNA-seq into cDNA using a reverse transcription kit (SM134, Saiwen Innovation (Beijing) Biotechnology Co., Ltd., Beijing, China) according to the MIQE guidelines (Bustin, 2009) [32]. The qPCR reactions were performed with three biological replicates per gene (the same individuals used for RNA-seq) and three technical replicates per biological replicate. We then performed the reactions on a QuantStudio 3 instrument in a 10 μL system containing 5 μL of 2× SYBR Green qPCR MasterMix II, 0.2 μL each of forward and reverse primers, 1 μL of cDNA template, and 3.6 μL of nuclease-free water.

The PCR protocol consisted of initial denaturation at 95 °C for 30 s, followed by 40 cycles of 95 °C for 15 s and annealing at the temperatures listed in Table 3 for 30 s. After amplification, we performed melting curve analysis according to the instrument’s recommended protocol. We calculated relative gene expression using the 2^−ΔΔCt^ method. Relative gene expression levels were calculated using the standard 2^−ΔΔCt^ method.

## 3. Results and Analysis

### 3.1. Selection of Optimal Growth Curve Models for Min Pigs

#### 3.1.1. Model Selection

Model comparison based on AIC and BIC strongly supported the Logistic growth function with individual-level random effects on all three parameters (k, a, b) as the best-fitting model (Table 4). This model had an AIC of 5823.3 and a BIC of 5873.6, which were 18.4 units lower than the next-best model (Gompertz with the same random-effects structure) and more than 400 units lower than models with random effects only on k. All mixed-effects models substantially outperformed the corresponding fixed-effects models, confirming the importance of accounting for individual heterogeneity. Models with additional batch-level random effects did not improve fit, indicating negligible batch-level variation.

#### 3.1.2. Parameter Estimates of the Final Model

Fixed-effect parameter estimates for the final model are presented in Table 5. The estimated asymptotic body weight (k) was 105.60 kg (SE = 1.63; 95% CI: 102.40–108.80). The timing parameter (a) was 21.55 days (SE = 0.85; 95% CI: 19.88–23.23), and the scale parameter (b) was 0.01849 (SE = 0.00034; 95% CI: 0.01782–0.01916). All fixed effects were highly significant (*p* < 0.0001).

The estimated random-effect variance–covariance matrix at the individual level is shown in Table 6. Substantial individual variability was observed for K (SD = 13.89 kg) and a (SD = 7.12 days), whereas variability in b was very small but statistically significant (SD = 0.00277; 95% CI: 0.00227–0.00338). The correlation between the random effects for a and b was strong (r = 0.78; 95% CI: 0.65–0.86), suggesting that pigs with a later inflection point tended to have a smaller scale parameter.

#### 3.1.3. Growth Characteristic Points

Based on the final model, we derived one inflection point and two characteristic transition points (Table 7). The early transition point, termed the Growth Rate Index (GRI), occurred at 94.83 days (body weight = 22.32 kg). The single inflection point, termed the Maximum Growth Rate (MGR), occurred at 166.06 days (body weight = 52.80 kg), and corresponded to a maximum absolute growth rate of 0.488 kg/day. The late transition point, termed the Late Growth Rate Index (LGRI), occurred at 237.29 days (body weight = 83.29 kg). These three points partitioned the growth trajectory into initial acceleration, rapid growth, deceleration, and Asymptotic growth phases.

#### 3.1.4. Model Diagnostics and Validation

**Figure A2:**
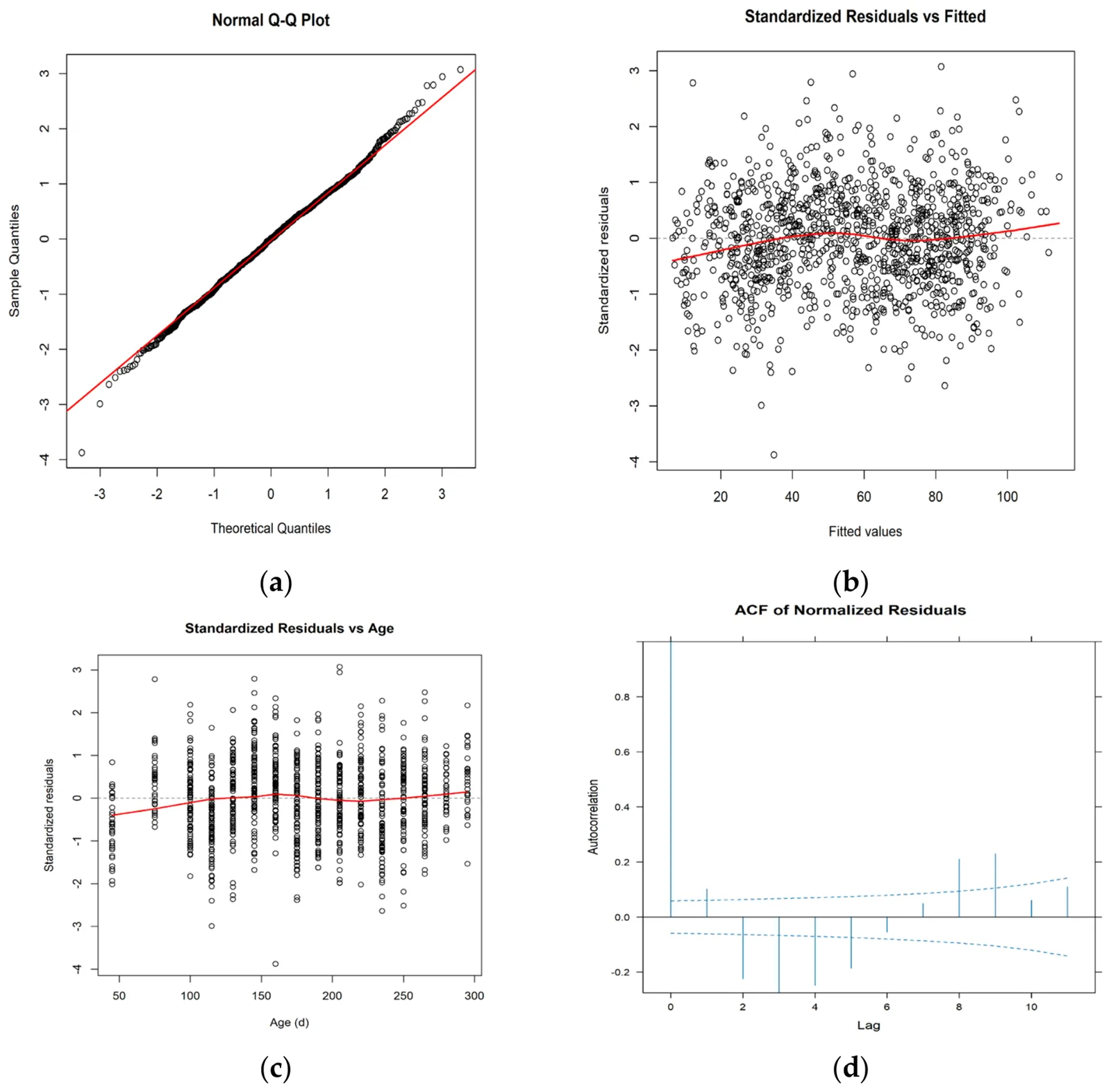
Residual diagnostic plots for the final Logistic nonlinear mixed-effects model. (**a**) Normal Q–Q plot of standardized residuals; (**b**) standardized residuals versus fitted values; (**c**) standardized residuals versus age; (**d**) autocorrelation function of normalized residuals. The plots show no marked deviations from normality, constant variance, or independence.

Model diagnostics and validation results are summarized in Table 8. The standardized residuals of the final model were approximately normally distributed (Shapiro–Wilk test: W = 0.9975, *p* = 0.083). The four diagnostic plots, including the Q–Q plot, standardized residuals versus fitted values, standardized residuals versus age, and the autocorrelation function, showed no evidence of heteroscedasticity, nonlinearity, or within-individual autocorrelation (Appendix A Figure A2). Models incorporating an additional continuous-time autoregressive correlation structure (corCAR1) or a power variance function (varPower) had higher BIC values than the final model (5880.6 vs. 5873.6), indicating that the simpler residual structure was adequate.

In leave-one-out cross-validation, the final model achieved an RMSE of 8.52 kg, which was slightly lower than that of the simpler model with only a random effect on K (8.58 kg). This indicates that the inclusion of random effects on a and b did not cause overfitting and that the final model provides a good balance between goodness-of-fit and predictive performance.

#### 3.1.5. Sensitivity Analyses

Sensitivity analyses were conducted to evaluate the potential influence of sex, family, batch, and unbalanced age coverage on growth parameter estimates (Appendix A Table A1 and Table A2). Including sex as a fixed effect on K or on all parameters did not improve model fit (AIC increased from 5823.3 to 5824.6 and 5827.2, respectively). Similarly, models with family or batch as random intercepts on K showed substantially higher AIC values (5900.7 for both), indicating that these effects were negligible.

Subset analyses provided further evidence for the robustness of the final model. Parameter estimates from the overlapping age subset (100–265 days) were highly consistent with those from the full dataset (K: 102.31 vs. 105.60 kg; a: 21.07 vs. 21.55 days; b: 0.01898 vs. 0.01849), with relative differences of less than 4%. Estimates from sex- and batch-specific subsets also differed by less than 10% from those of the full model, confirming that the growth parameters were not materially biased by unbalanced age coverage or by sex and batch effects.

### 3.2. Transcriptome Analysis of Longissimus Dorsi Muscle in Min Pigs with Different Growth Rates

#### 3.2.1. Sequencing Data Statistics

RNA-seq of the 10 growth-rate samples generated 71.62 Gb of clean data, with each sample yielding at least 6.15 Gb. The Q30 percentage for all samples was ≥97.96%, indicating high sequencing quality. The mapping efficiency against the pig reference genome (Sus scrofa, assembly Sscrofa11.1) ranged from 89.34% to 96.63% (Table 9), confirming reliable alignment and suitability for downstream analyses. The distribution of gene expression levels across all samples was comparable after normalization (Supplementary Figure S1).

#### 3.2.2. Differential Expression Gene Analysis

**Figure 1:**
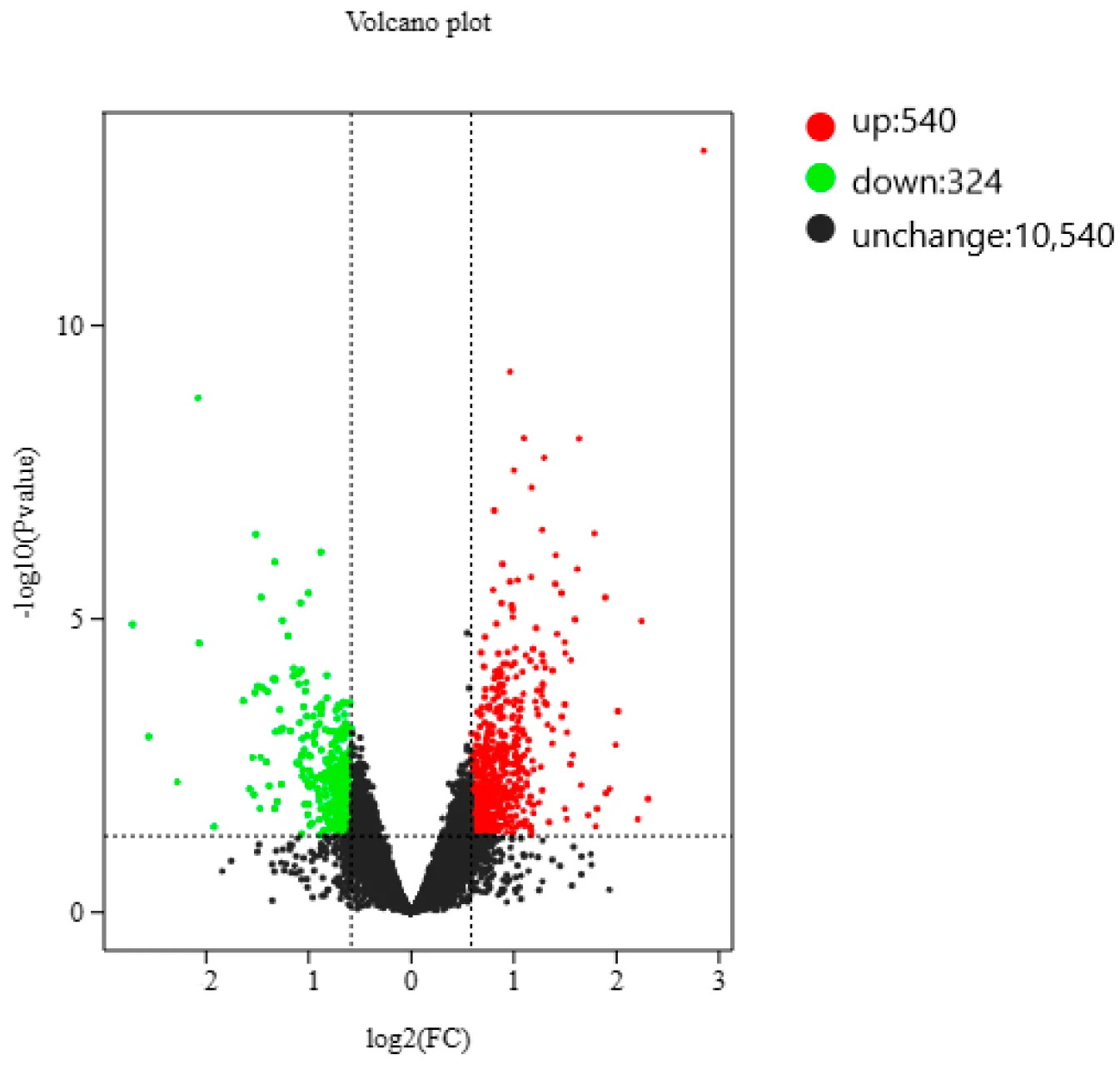
Volcano plot of DEGs in Min pig growth rate.

Using DESeq2 (Love, 2014) [33], we screened for DEGs between the rapid and slow growth groups based on raw count data, applying thresholds of |fold change| ≥ 1.5 and *p*-value < 0.05. We identified 864 DEGs, of which 540 were upregulated and 324 downregulated. To further control the false discovery rate, we additionally applied Benjamini–Hochberg FDR correction (q < 0.05) to the same dataset, and 189 genes remained significant under this more stringent criterion, indicating that our analysis results are highly robust. Figure 1 presents a volcano plot visualizing the distribution of these 864 DEGs. Principal component analysis (PCA) revealed a clear separation between the fast- and slow-growing groups along the first principal component (Supplementary Figure S2). Hierarchical clustering of all samples based on gene expression profiles further confirmed that samples clustered primarily by growth phenotype (Supplementary Figure S3).

#### 3.2.3. Enrichment Analysis of Differentially Expressed Genes

**Figure 2:**
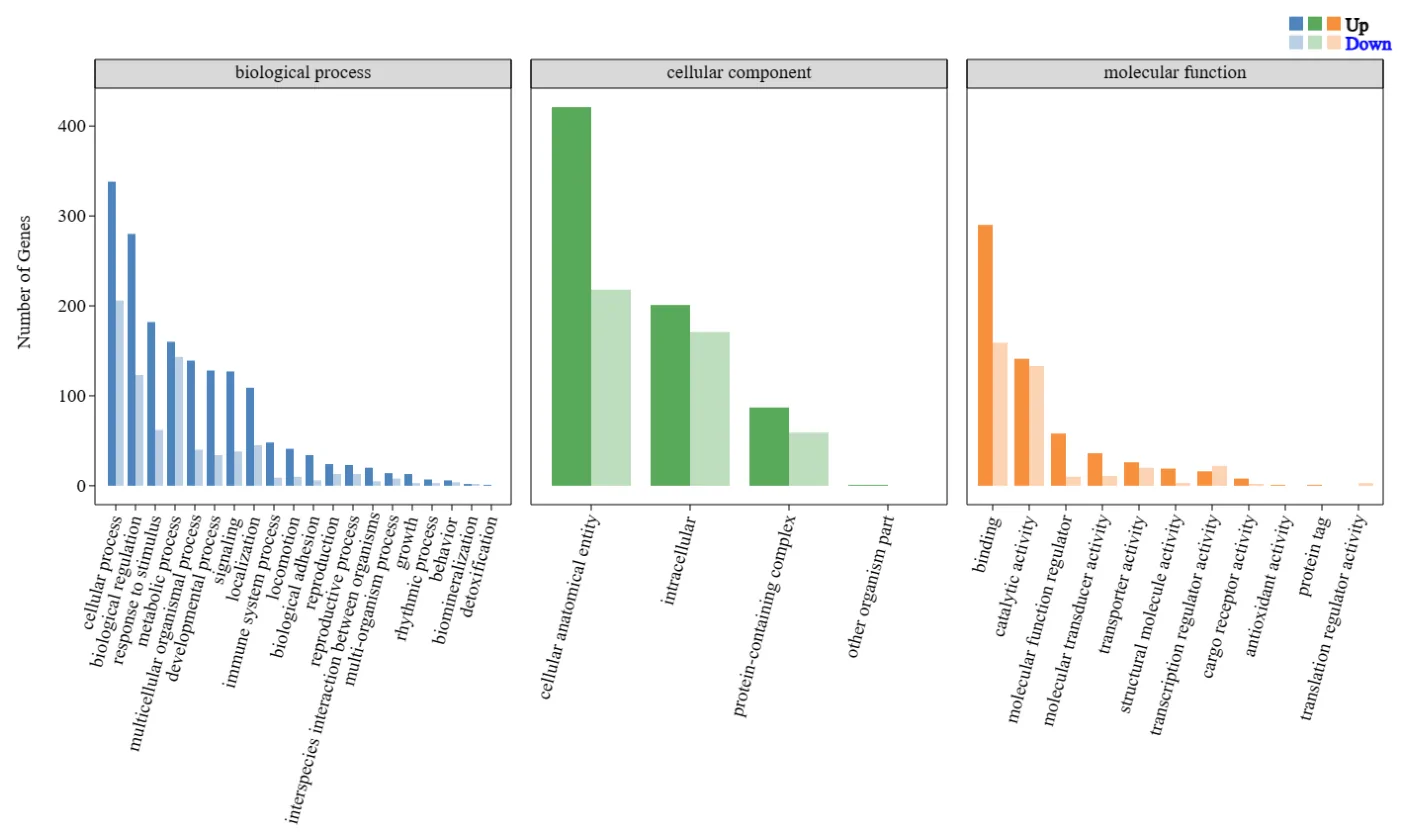
GO enrichment of DEGs in Min pig growth rate.

To explore functional genes and signaling pathways involved in growth regulation, we performed GO and KEGG enrichment analyses on the 864 DEGs. Of these, 801 genes were successfully annotated in the GO database (Figure 2) and assigned to 20 biological process (BP), 11 cellular component (CC), and 4 molecular function (MF) subcategories.

Among the top 20 GO terms (ranked by *p*-value), the DEGs were predominantly enriched in BP terms including extracellular matrix organization, multicellular organism development, and cell adhesion (Supplementary Figure S4); CC terms such as extracellular space and endoplasmic reticulum; and MF terms such as extracellular matrix structural constituent, heparin binding, and integrin binding.

Notably, the DEGs showed significant enrichment in growth-related BP terms, including the transforming growth factor-β receptor signaling pathway (GO:0007179), integrin-mediated signaling pathway (GO:0007229), oxidative phosphorylation (GO:0006119), skeletal muscle tissue development (GO:0007519), and collagen fibril organization (GO:0030199). Enriched MF terms included calcium ion binding (GO:0005509), collagen binding (GO:0005518), and protein binding (GO:0005515). Enriched CC terms included myofibril (GO:0030016), mitochondrion (GO:0005739), type III collagen trimer (GO:00055586), mitochondrial inner membrane (GO:0005743), and actin cytoskeleton (GO:0015629).

**Figure 3:**
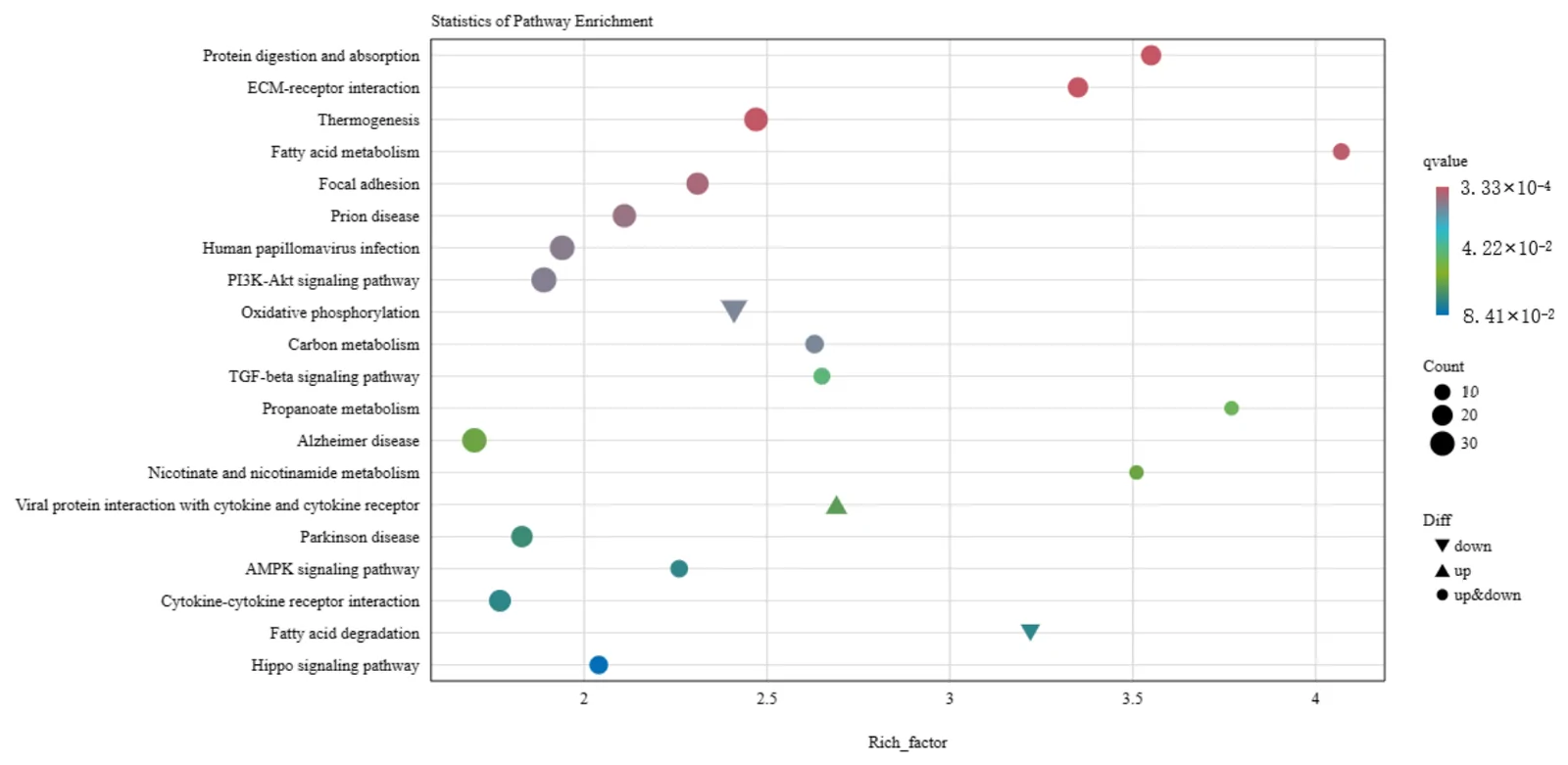
KEGG pathway enrichment of DEGs in Min pig growth rate.

To further elucidate the molecular basis of growth rate divergence in pigs, we performed hypergeometric tests for pathway enrichment among the DEGs and visualized the results with ClusterProfiler (Yu, 2012) [34]. Figure 3 presents a bubble plot of the KEGG enrichment results, showing the top 20 pathways ranked by q-value. The DEGs were predominantly enriched in pathways related to skeletal muscle development, thermogenesis, lipid metabolism, and immunity. Notably, the PI3K-Akt, AMPK, and TGF-β signaling pathways emerged as core regulators of porcine growth performance, consistent with their established roles in muscle development. The complete lists of GO and KEGG enrichment results are provided in Supplementary Table S1.

#### 3.2.4. RT qPCR Validation Results

**Figure 4:**
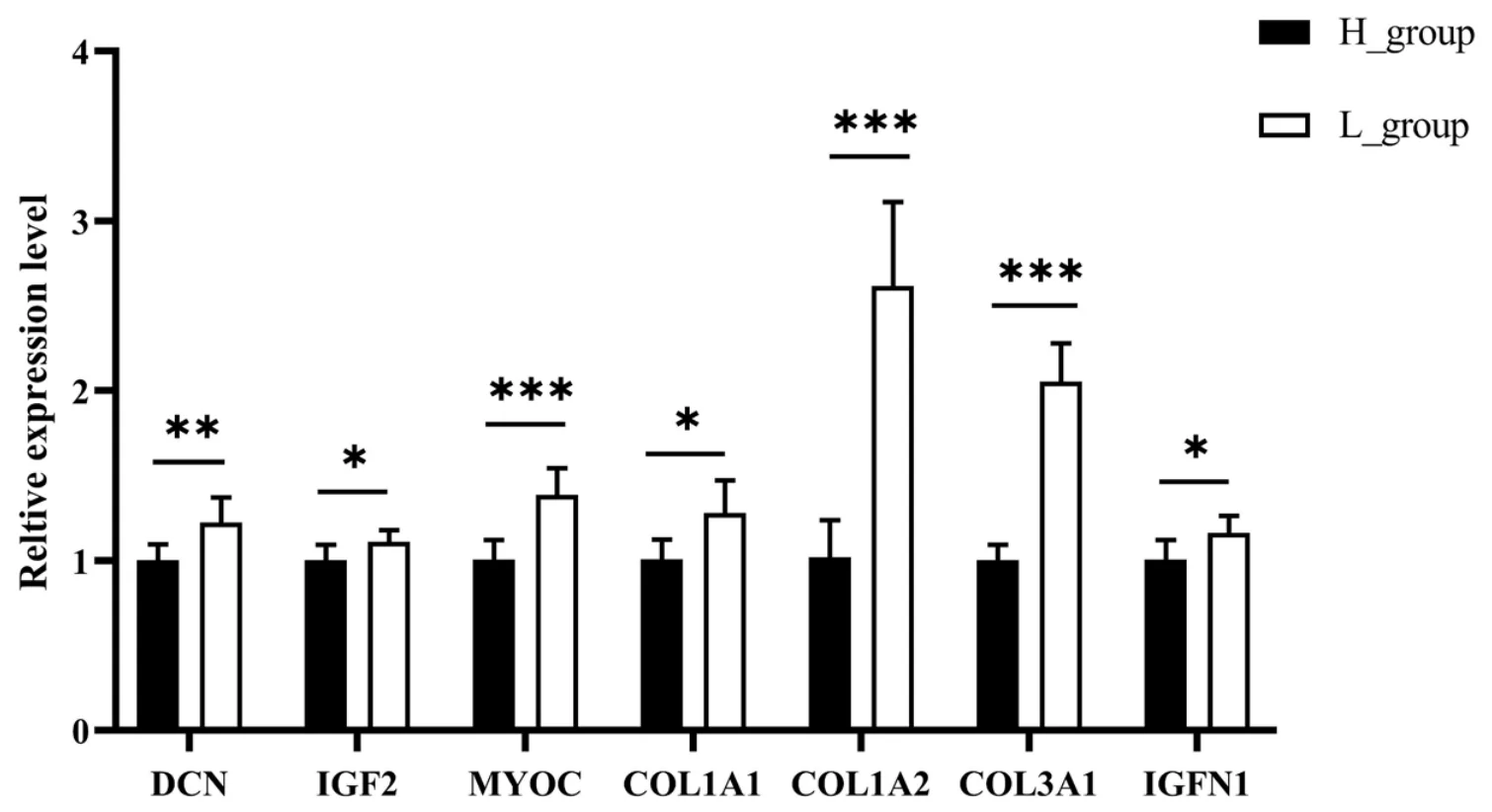
RT qPCR validation of growth rate group. Notes: *: *p* < 0.05, ** *p* < 0.01, ***: *p* < 0.001, versus control group.

We validated the RNA-seq data using quantitative RT-qPCR. From the transcriptome data, we randomly selected seven DEGs and used GAPDH as the reference gene. We performed RT-qPCR on the same longissimus dorsi muscle RNA samples used for RNA-seq (Figure 4). The RT-qPCR results largely agreed with the RNA-seq data, confirming the reliability of the transcriptome data and its suitability for downstream analyses.

## 4. Discussions

### 4.1. Selection of Optimal Growth Curve Models

To fit Min pig growth curves, we compared nonlinear fixed-effects models and nonlinear mixed-effects models with different random-effect structures based on the Gompertz, Logistic, and von Bertalanffy functions. Among the fixed-effects models, the Logistic model performed best, with the lowest AIC and BIC and the highest log-likelihood. This is consistent with previous findings in Pudong White pigs [35] and Qingyu pigs [36], supporting the general utility of the Logistic function for describing growth in Chinese indigenous pig breeds.

Fixed-effects models provide reasonable parameter estimates when the population is relatively homogeneous. However, when individual variation is substantial, these models may lack robustness because they ignore individual differences and the hierarchical structure of repeated measurements [23,35,36]. In our data, all mixed-effects models clearly outperformed the fixed-effects models, as indicated by lower AIC and BIC and higher log-likelihood values. This result is in agreement with previous studies showing that mixed-effects models improve fit by accounting for individual heterogeneity [35,36].

We further compared mixed-effects models with varying random-effect structures. Although models with more complex hierarchical structures can theoretically capture additional sources of variation [37], overly complex random-effect specifications may lead to overfitting and reduced interpretability [23]. In our analysis, the best model was the Logistic nonlinear mixed-effects model with individual-level random effects on all three growth parameters (K, a, and b). This model had the lowest AIC (5823.3) and BIC (5873.6) among all candidate models and clearly outperformed simpler mixed-effects models with random effects only on K. Adding batch-level random effects did not improve fit, indicating that batch variation was negligible after accounting for individual-level heterogeneity.

Sensitivity analyses further supported the robustness of this model choice. Adding sex as a fixed effect on K or on all parameters did not reduce AIC or BIC, and models including family or batch as random intercepts on K showed substantially higher AIC values, indicating that these potential confounders had negligible effects. In addition, growth parameters estimated from the overlapping age subset (100–265 days) were highly consistent with those from the full dataset, with relative differences of less than 4%, confirming that the unbalanced age coverage between batches did not materially bias the model estimates. These results collectively demonstrate that the selected model was stable and not driven by sex, family, batch, or age-range imbalance.

In conclusion, the Logistic nonlinear mixed-effects model with individual-level random effects on all three growth parameters represents the optimal choice for fitting Min pig growth curves. Compared with fixed-effects models and simpler mixed-effects models, it provides more precise population-level estimates and better characterizes individual growth trajectories.

### 4.2. Analysis of Growth Curve Parameters

Based on the final Logistic nonlinear mixed-effects model, the estimated asymptotic body weight (K) of Min pigs was 105.60 kg. This value is lower than those reported for Yorkshire [38], Liangshan pigs [39], and Brazilian castrated boars [40], but remains comparable to values reported for Pudong White boars [35]. Min pigs reached the inflection point of maximum absolute growth rate (MGR) at 166.06 days, with a corresponding body weight of 52.80 kg. In comparison, Vietnamese indigenous Tap Na sows peaked at 185.1 days (48.29 kg) [41], Pudong White boars at 198.24 days (46.36 kg) [35], and Liangshan pigs at 193.4 days (62.5 kg). These comparisons indicate that Min pigs reached their maximum growth rate earlier than several other indigenous breeds, but with a moderate inflection-point weight and a relatively low mature weight.

Earlier attainment of maximum growth rate may be related to the developmental sequence of tissues. Previous evidence indicates that the sequence of tissue maturation in Min pigs is bone, muscle, skin, and fat, whereas in lean-type breeds such as Landrace, the sequence is bone, skin, muscle, and fat [42]. The relatively early muscle development in Min pigs may therefore contribute to their earlier peak growth rate. In addition, Min pigs possess well-developed shoulder muscles but relatively underdeveloped hindlimb muscles, which may partly explain their lower body weight at the maximum growth point. Differences in model selection may also contribute to variation in reported growth parameters across breeds [39].

Using the same model, we derived one inflection point and two characteristic transition points: the early transition point (Growth Rate Index, GRI) at 94.83 days (22.32 kg), the inflection point (MGR) at 166.06 days (52.80 kg), and the late transition point (Late Growth Rate Index, LGRI) at 237.29 days (83.29 kg). Based on these points, the growth trajectory of Min pigs was divided into four successive phases: initial acceleration (before approximately 95 days), rapid growth (approximately 95–166 days), deceleration (approximately 166–237 days), and asymptotic growth (after approximately 237 days). During the asymptotic growth phase, body weight continued to increase but at a progressively declining rate, approaching the asymptotic value.

From a practical perspective, the initial acceleration phase is a critical period for piglet health and early development, and it directly influences subsequent growth performance. The rapid growth phase represents the period of greatest daily gain, during which nutritional and management interventions have the largest impact on overall growth. The deceleration and asymptotic growth phases are important for determining optimal slaughter timing, because growth rate declines and feed efficiency decreases as animals approach mature weight. Therefore, stage-specific feeding and management strategies should be considered to fully exploit the growth potential of Min pigs.

### 4.3. Study on Gene Differential Expression of Growth Performance in Min Pigs

Vinkel and Unger emphasized that rigorous experimental design is essential for obtaining reliable DEG profiles in transcriptome studies of growth regulation in pigs [43,44]. Gender and growth stage, as two core biological variables, systematically influence gene expression. Xie demonstrated that distinct developmental phases, namely slow growth, rapid growth, deceleration, and plateau, correspond to different physiological states and metabolic centers, with marked differences between successive stages [45]. Accordingly, we restricted all samples to the same gender and to a single developmental stage, which we determined from our growth curve fitting results. This design aligns with the principles advocated by Li et al. and Randell et al. [46,47]. By controlling for gender and growth stage as confirmed key confounding factors, we standardized the physiological context, allowing a more accurate interpretation of the molecular mechanisms underlying individual phenotypic differences. Specifically, we selected samples from the plateau stage for comparative analysis to facilitate the identification of genes and pathways involved in growth, development, metabolic homeostasis, and growth potential during the deceleration stage of Min pigs.

A total of 864 DEGs were identified between the fast- and slow-growing groups via transcriptome sequencing, with 540 upregulated and 324 downregulated in the fast-growing group. GO and KEGG enrichment analyses revealed seven genes that were showed differential expression trends in the fast growth group: *IGFN1*, *DCN*, *COL3A1*, *MYOC*, *COL1A1*, *COL1A2*, and *IGF2*. Notably, under the more stringent FDR-corrected threshold (q < 0.05), only *IGFN1* exhibited a trend toward differential expression (log2FC = –1.51, *p* = 0.09, q = 0.47), whereas the remaining six genes did not reach statistical significance. This may be attributed to the limited statistical power associated with the small sample size (n = 5 per group), as well as potential differences in reference genome annotation or tissue-specific expression patterns. It is important to emphasize that these genes were initially identified based on unadjusted *p*-values (*p* < 0.05), and their differential expression was further corroborated by RT-qPCR validation within the same cohort. Thus, while the expression differences observed for these candidate genes are considered reliable in the context of the present study, larger independent cohorts will be essential to establish genome-wide FDR significance for these specific transcripts. Nevertheless, their consistent expression patterns and established roles in muscle development and ECM remodeling support their potential involvement in growth regulation, warranting further investigation in larger cohorts. Based on their functional roles, we classified these genes into three potential modules: the ECM-related collagen family, the *IGF2* signaling axis, and the myogenesis/cytoskeletal module.

#### 4.3.1. ECM Remodeling as Structural Support for Growth

In fast-growing Min pigs, coordinated upregulation of COL1A1 and COL1A2 was observed, whereas COL3A1 showed differential expression patterns between the two groups. This synchronized increase in the two type I collagen genes suggests that the fast-growing group may have enhanced ECM scaffold remodeling in association with rapid muscle accretion. In contrast, the stage-dependent modulation of *COL3A1* points to a role in fine-tuning ECM elasticity and assembly dynamics during tissue expansion. Notably, the upregulation of type I collagens was associated with the fast-growing phenotype in the present cross-sectional comparison. These expression patterns are consistent with the hypothesis that ECM remodeling may actively support muscle growth; however, this interpretation is based on cross-sectional data and requires longitudinal validation. Such coordinated transcriptional activation may represent a fundamental strategy by which rapidly growing muscle maintains both mechanical integrity and adaptive capacity within its connective tissue framework.

Collagens are major structural components of the ECM that maintain tissue integrity and support myogenesis [44,45]. The α1 and α2 chains encoded by *COL1A1* and *COL1A2* form the most abundant heterotrimer in skeletal muscle ECM, providing mechanical support for myocyte proliferation and tissue organization. Previous studies have linked *COL3A1* variants to body length in Meishan pigs and growth traits in cattle [46,47], supporting its involvement in growth regulation. Our findings suggest that type I and type III collagens may be differentially regulated in association with rapid muscle growth, rather than serving as passive structural elements. The upregulation of *COL1A1* and *COL1A2* may enhance ECM stiffness and tensile strength, whereas the differential expression of COL3A1 may provide flexibility for ECM remodeling.

This expression pattern aligns with previous reports on stage-specific collagen expression [48,49,50] and parallels observations in bovine skeletal muscle, suggesting an evolutionarily conserved mechanism by which ECM components facilitate rapid muscle growth. Overall, our data indicate that ECM-related genes in fast-growing pigs constitute a structural support module that actively responds to growth signals and provides the mechanical foundation for rapid muscle expansion, complementing the anabolic and myogenic programs discussed in the following sections.

#### 4.3.2. IGF2 in Anabolic Drive for Growth

In fast-growing Min pigs, *IGF2* transcript levels were significantly elevated, positioning it as a primary candidate associated with the enhanced growth phenotype. Mechanistically, *IGF2* may exert its anabolic effects through the PI3K-Akt cascade: it may promote phosphorylation of PKB and IGF1R, upregulate myogenic differentiation factors, accelerate myocyte differentiation, and enhance protein synthesis and muscle hypertrophy [13,14]. It also regulates intramuscular fat deposition via the PI3K-Akt/AMPK axis [51] and interacts with TGF-β signaling during myogenesis.

The elevated IGF2 expression we observed aligns with findings in commercial pig breeds, where a G-to-A substitution in intron 3 disrupts ZBED6 binding and increases IGF2 mRNA levels approximately threefold [52,53]. While the specific regulatory variant in Min pigs remains unknown, the convergence of our data with these established effects supports *IGF2*-driven anabolism as a conserved determinant of growth. Our findings provide the first evidence that this pathway is active in the rapid growth phenotype of Min pigs. Collectively, *IGF2* may represent an anabolic module associated with muscle growth, potentially linking to PI3K-Akt signaling, energy metabolism, and ECM remodeling pathways. In the present study, its elevated expression in the fast-growing group suggests a potential association with these processes.

#### 4.3.3. IGFN1 and MYOC in Myogenesis and Cytoskeletal Stabilization

In fast-growing Min pigs, *IGFN1* and *MYOC* were significantly upregulated in skeletal muscle, pointing to coordinated enhancement of myogenic differentiation and cytoskeletal dynamics. *IGFN1* maintains myocyte structural stability and regulates protein synthesis [54,55], with functional evidence linking *MSTN* signaling to its expression [56,57]. *MYOC* has been reported to participate in cytoskeletal assembly and signal transduction, and to affect MAPK, Wnt, and TGF-β pathways, as well as to promote proliferation and inhibit apoptosis via ERK1/2-MAPK [58,59,60,61].

The complementary actions of *IGFN1* and *MYOC* likely facilitate rapid growth through a dual mechanism: *IGFN1* enhances structural stability and protein synthesis, whereas *MYOC* promotes proliferation and inhibits apoptosis, providing a cellular pool for muscle accretion. These findings are consistent with mouse models [55,56,61] and extend this regulatory axis to porcine growth. Collectively, *IGFN1* and *MYOC* form a myogenesis and cytoskeletal stabilization module that provides the cellular foundation for rapid muscle growth, complementing the ECM structural support and *IGF2*-driven anabolism.

#### 4.3.4. Synergistic Regulatory Network and Integrated Model

The three modules described above, namely ECM structural support, *IGF2*-driven anabolism, and *IGFN1/MYOC*-mediated myogenesis, do not operate independently but form an integrated network. At the signaling level, *IGF2*-activated PI3K-Akt may indirectly upregulate ECM genes via transcription factors such as SP1 and CTGF. At the functional level, *DCN* complements the *IGFN1–MSTN* axis by inhibiting TGF-β signaling, thereby relieving *MSTN*-mediated suppression of muscle growth. At the structural level, coordinated ECM gene expression supports their co-assembly into a stable collagen network.

Beyond the fast-growing modules, the slow-growing group showed enrichment of oxidative phosphorylation, lipid metabolism, and immune pathways, suggesting a trade-off between rapid growth and other physiological demands. Taken together, our findings support a working model in which *IGF2*-driven anabolism supplies metabolic fuel, ECM-mediated support provides the physical scaffold, and *IGFN1/MYOC*-regulated myogenesis ensures cellular supply. These three complementary pillars synergistically support the superior growth of fast-growing Min pigs.

In contrast to the fast-growing group, the slow-growing group showed significant enrichment of oxidative phosphorylation, lipid metabolism, and immune-related pathways. Several mitochondrial genes (*MT-CO3*, *MT-ATP6*, *MT-ND4*, and *CYTB*) were highly expressed in this group. However, elevated mitochondrial transcript levels may reflect increased mitochondrial abundance, fiber type composition, or technical variation, and do not necessarily indicate enhanced mitochondrial activity. Additional measurements (e.g., mitochondrial DNA copy number, protein levels, or enzymatic activity) are required to confirm functional changes in mitochondrial activity. Mitochondrial DNA has been widely used to study genetic structure in animal populations due to its high mutation rate and maternal inheritance [62], and future work should examine whether mitochondrial variants contribute to growth variation in Min pigs. We also identified lipid metabolism genes (*PHYH*, *FABP3*, *ACADVL*, *S100A1*, *HADHB*) overexpressed in the slow-growing group, along with immune-related DEGs. Given that slow-growing pigs have greater backfat thickness at advanced ages, this may explain the enrichment of immune pathways, as adipose tissue functions as an endocrine and immune organ that regulates metabolism and immunity through peptide secretion [63].

Several limitations should be acknowledged. The sample size was small (five pigs per group), and the findings require validation in larger populations. The study relied solely on transcriptome data without protein-level confirmation. The functional roles of *IGFN1*, *MYOC*, and the regulatory network involving collagens and *IGF2* signaling warrant further investigation. Future studies may explore whether the candidate genes identified here are associated with heritable DNA variants for marker-assisted selection, and whether nutritional modulation of collagen-synthesis nutrients could influence muscle development.

## 5. Conclusions

The Logistic nonlinear mixed-effects model with individual-level random effects on all three growth parameters was identified as the optimal model for fitting Min pig growth curves. From this model, one inflection point and two characteristic transition points were derived: the early transition point (Growth Rate Index, GRI) at 94.83 days (22.32 kg), the single inflection point (Maximum Growth Rate, MGR) at 166.06 days (52.80 kg), and the late transition point (Late Growth Rate Index, LGRI) at 237.29 days (83.29 kg). The maximum absolute growth rate was 488.2 g/day. These points partitioned the growth trajectory into four successive phases: initial acceleration, rapid growth, deceleration, and Asymptotic growth. Furthermore, transcriptome analysis revealed that the superior growth of fast-growing Min pigs is driven by the coordinated upregulation of seven genes—*IGFN1*, *DCN*, *COL3A1*, *MYOC*, *COL1A1*, *COL1A2,* and *IGF2*—which converge on the PI3K-Akt, AMPK, and TGF-β signaling pathways. This gene module, together with the FDR-validated high-confidence DEGs, provides a multilayered molecular framework for understanding growth variation in indigenous breeds and highlights candidate targets for future functional validation and genetic improvement studies.

## Figures and Tables

**Table 1:** Inflection point parameters for estimating three types of nonlinear functions.

Parameter	Gompertz	Logistic	Von Bertalanffy
$t_{GRI}$	ln0.382a/b	$\left(\ln a-1.317\right)/b$	ln1.354a/b
$W_{GRI}$	$k/e^{1/0.382}$	$k/\left(1+e^{1.317}\right)$	0.0179k
$t_{MGR}$	lna/b	lna/b	ln3a/b
$W_{MGR}$	ke	k/2	8k/27
$t_{LGRI}$	ln2.618a/b	$\left(\ln a+1.317\right)/b$	ln6.646a/b
$W_{LGRI}$	$k/e^{1/2.618}$	$ke^{1.317}/\left(1+e^{1.317}\right)$	0.613k
$V_{max}$	kb/e	kb/4	4kb/9

Note: “k” indicates the Asymptotic (maximum mature) weight; “a” indicates the time parameter; “b” indicates the be parameter controlling growth rate as weight approaches maturity; “GRI” indicates the early transition point from the initial acceleration phase to the rapid growth phase (Growth Rate Index); “MGR” indicates the inflection point of maximum absolute growth rate (Maximum Growth Rate); “LGRI” indicates the late transition point from the rapid deceleration phase to the Asymptotic growth phase (Late Growth Rate Index); “tGRI” indicates the age at the early transition point; “WGRI” indicates the body weight at the early transition point; “tMGR” indicates the age at the inflection point of maximum growth rate; “WMGR” indicates the body weight at the inflection point of maximum growth rate; “tLGRI” indicates the age at the late transition point; “WLGRI” indicates the body weight at the late transition point; “Vmax” indicates the maximum absolute growth rate (maximum daily weight gain).

**Table 2:** Phenotypic information statistics of transcriptome samples.

Groups	*n*	Slaughter Age	Slaughter Weight (kg)
Fast-growing	5	240.5 ± 20.42	90
Slow-growing	5	286.67 ± 19.2 **	90

Note: ** *p* < 0.01 compared with the fast-growing group (Independent samples Student’s *t*-test: *t* = 3.682, df = 8, two-tailed *p* = 0.006). Data are expressed as mean ± SD.

**Table 3:** Primers for Real-time PCR.

Genes	Primer Sequences (5′→ 3′)		Annealing Temperature/°C	Product Length/bp
DCN	F:	GGCAACTATCATCTTCCTCT	53.5	215
	R:	TCCAGACCCAAATCAGAAC		
IGF2	F:	GTGCTGCTATGCTGCTTA	53	105
	R:	GGCCTGCTGAAGTAGAAG		
MYOC	F:	GCTCCAGGGAAGTTTCTA	51.5	206
	R:	CTTGCCAGTGATTGTCTC		
COL1A1	F:	AATGTGTTGTGCGATGAC	53.5	282
	R:	AGCAAAGTTTCCTCCGAG		
COL1A2	F:	GCCGAGGGCAACAGCAGATT	49.6	123
	R:	AAGGATAGGCAGGCGAGATG		
COL3A1	F:	AACAGGAAGCTATTGAAGGA	52	129
	R:	ATATTATATCATCGCAGAGAACAG		
IGFN1	F:	GGTCGTCCCAAAGGAGCAG	58.1	112
	R:	CAGCAGGTGGGTCCGTAGC		
GAPDH	F:	CCCCAACGTGTCGGTTGT	55	83
	R:	CCTGGTTCACCACCTCTTCTGA		

**Table 4:** Comparison of candidate nonlinear mixed-effects models for body weight growth.

Growth Function	Random Effects	df	logLik	AIC	BIC	∆AIC	∆BIC
Logistic	k + a + b~1	10	−2902	5823	5874	0	0
Gompertz	k + a + b~1	10	−2911	5842	5892	18.41	18.41
Gompertz	k + a~1	7	−2951	5915	5950	91.88	76.81
Logistic	k + a~1	7	−2974	5962	5997	138.28	123.21
Von_Bertalanffy	k + a~1	7	−2995	6003	6039	180.04	164.96
Gompertz	k~1	5	−3110	6230	6255	406.77	381.65
Logistic	k~1	5	−3110	6230	6255	406.88	381.76
Gompertz	k~1|Batch/ID	6	−3110	6232	6262	408.24	388.14
Logistic	k~1|Batch/ID	6	−3110	6232	6262	408.58	388.49
Von_Bertalanffy	k~1	5	−3142	6294	6319	470.56	445.44
Von_Bertalanffy	k~1|Batch/ID	6	−3142	6295	6325	471.93	451.83
Logistic	None (fixed effects)	4	−3942	7891	7912	2068.12	2037.98
Gompertz	None (fixed effects)	4	−3956	7921	7941	2097.18	2067.04
Von_Bertalanffy	None (fixed effects)	4	−3968	7944	7964	2120.91	2090.77

Notes: ΔAIC and ΔBIC are differences relative to the best-fitting model. All mixed-effects models were fitted by maximum likelihood (ML). Models with batch-level random effects and all three parameters (e.g., k + a + b|Batch/ID) were tested but had higher AIC/BIC.

**Table 5:** Fixed-effect parameter estimates for the final Logistic nonlinear mixed-effects model.

Parameter	Estimate	SE	df	*t*-Value	*p*-Value	95% CI
k	105.6003	1.6309	1119	64.75	<0.0001	[102.401, 108.800]
a	21.551	0.8545	1119	25.22	<0.0001	[19.875, 23.227]
b	0.01849	0.00034	1119	54.24	<0.0001	[0.01782, 0.01916]

Notes: “k” indicates the maximum mature weight; “a” indicates the time parameter; “b” indicates the growth rate when it tends to mature; 95% confidence intervals were obtained using the intervals() function in the nlme package.

**Table 6:** Estimated random-effect variance–covariance matrix at the individual level for the final model.

Random Effect	SD	Variance	95% CI for SD	Correlations
k	13.888	192.875	[11.531, 16.727]	—
a	7.124	50.752	[5.749, 8.828]	r(k, a) = 0.096 [−0.161, 0.341]
b	0.00277	7.676 × 10^−6^	[0.00227, 0.00338]	r(k, b) = −0.224 [−0.457, 0.037]
				r(a, b) = 0.777 [0.652, 0.861]
Residual	2.275	5.178	[2.168, 2.388]	—

Notes: Random effects were specified at the individual level with an unstructured variance–covariance matrix (Log-Cholesky parameterization). Confidence intervals for correlations are approximate 95% intervals.

**Table 7:** Population-level growth curve inflection point parameters derived from the fixed effects of the final Logistic nonlinear mixed-effects model.

t_GRI_/d	t_MGR_/d	t_LGRI_/d	W_GRI_/kg	W_MGR_/kg	W_LGRI_/kg	V_max_/kg/d
94.83	166.06	237.29	22.32	52.8	83.29	0.4881

Notes: tGRI, tMGR, and tLGRI represent the ages at which absolute growth rate reaches 25%, 100%, and 75% of its maximum value (Vmax), respectively. WGRI, WMGR, and WLGRI are the corresponding body weights at those ages. Vmax is the maximum absolute growth rate.

**Table 8:** Model diagnostics and validation for the final Logistic nonlinear mixed-effects model.

Item	Test/Model	Statistic	Value
Normality of standardized residuals	Shapiro–Wilk test	W	0.9975
		*p*-value	0.083
Residual autocorrelation	Final model (no correlation structure)	BIC	5873.6
	Model with corCAR1	BIC	5880.6
	Model with varPower	BIC	5880.6
Batch-level random effect	Final model (no Batch)	AIC	5823.3
	Model with k + a + b|Batch/ID (diagonal)	AIC	5896.8
		BIC	5947
Predictive performance (LOOCV)	Final model	RMSE (kg)	8.5155
	Simpler model (k only)	RMSE (kg)	8.5803

Notes: LOOCV = leave-one-out cross-validation; RMSE = root mean squared error. For the LOOCV, predictions were made using the fixed-effects part only (level = 0).

**Table 9:** Growth rate transcriptome data quality control and sequence alignment results statistics.

Samples	Total Reads	Mapped Reads	Q20(%)	Q30(%)
Age-H1	57,047,868	54,778,439 (96.02%)	99.8	98.86
Age-H6	47,872,628	46,138,434 (96.38%)	99.7	98.32
Age-H3	43,194,870	41,672,774 (96.48%)	99.63	97.96
Age-H4	41,862,698	40,283,156 (96.23%)	99.68	98.21
Age-H5	41,042,750	39,660,466 (96.63%)	99.68	98.23
Age-L1	57,153,296	54,820,906 (95.92%)	99.73	98.46
Age-L2	46,251,058	44,638,848 (96.51%)	99.72	98.37
Age-L3	45,795,036	43,979,374 (96.04%)	99.76	98.62
Age-L4	48,754,290	46,884,041 (96.16%)	99.66	98.03
Age-L5	49,103,312	47,443,685 (96.62%)	99.68	98.12

Note: samples: sample analysis number; Total reads: number of clean reads, calculated by single end; Mapped reads: the number of reads aligned to the reference genome and its percentage in clean reads; Q20 (%): percentage of bases with base recognition accuracy above 99%; Q30 (%): the percentage of bases with base recognition accuracy above 99.9%.

## Data Availability

The raw RNA-seq reads are not publicly available at this time due to ongoing project requirements. The data can be obtained from the corresponding author upon reasonable request.

## Supplementary Materials

The following supporting information can be downloaded at: https://www.mdpi.com/article/10.3390/ani16172783/s1.

## Appendix A

The R code for the nonlinear fixed-effects model and the nested two-level nonlinear mixed-effects model is given below as an example of the Gompertz function. Comments are provided that describe the specific details of the procedures used. The R code explanations are given by #.

The following code is for R implementation.

**Table A1:** Model comparison for the inclusion of sex, family, and batch effects in the final Logistic nonlinear mixed-effects model.

Model	AIC	BIC	∆AIC
Final (base)	5823.33	5873.57	0
Sex on Asym	5824.58	5879.84	1.25
Sex on all parameters	5827.18	5892.49	3.85
Family fixed (Asym)	5826.27	5911.67	2.94
Family random (Asym)	5900.74	5940.93	77.41
Batch random (Asym)	5900.74	5940.93	77.41

Notes: ∆AIC is the difference in AIC relative to the final base model. Lower AIC/BIC indicates better fit. The final base model is the Logistic model with individual-level random effects on K, a, and b. Family random and batch random models showed identical fit because family and batch were highly confounded in the data.

**Table A2:** Sensitivity analysis of growth curve parameters estimated from different data subsets.

Dataset	K (kg)	a (d)	b (d^−1^)	Model Type
Full combined	105.6	21.55	0.01849	nlme (full RE)
Male only	101.7	19.78	0.01855	nlme (K RE) ^1^
Female only	105.69	21.88	0.01886	nlme (K RE) ^1^
Batch 1 only	103.6	19.56	0.01802	nlme (K RE) ^1^
Batch 2 only	104.19	22.23	0.0195	nlme (K RE) ^1^
Overlap (100–265 d)	102.31	21.07	0.01898	nlme (K RE) ^1^

Notes: ^1^ Due to reduced sample sizes, simplified random-effect structures or fixed-effects models were used where necessary. The overlapping age subset completely avoids extrapolation outside the common age range of the two batches.
